# Supplementary material for: Genome-wide association study revealed genomic regions associated with tuber quality traits in water yam (Dioscorea alata L.)
Source: PLoS One. 2026 Feb 4;21(2):e0339978. doi: 10.1371/journal.pone.0339978 (PMC12871974; doi:10.1371/journal.pone.0339978)
Supplement: S1 Table — (DOCX) [file pone.0339978.s001.docx]

**S1 Table.** SNP markers associated with tuber oxidative browning in water yam

| Trait name | Model | Method | Marker | Chrom | Pos | QTN effect | LOD score | r2 (%) | MAF | Allele |
| --- | --- | --- | --- | --- | --- | --- | --- | --- | --- | --- |
| TOxB | Naive | pKWmEB | Chr8_508116 | 8 | 508116 | -0.0003 | 3.6623 | 2.0059 | 0.3246 | A |
|  |  | mrMLM | Chr18_3861135 | 18 | 3861135 | -0.2775 | 3.113 | 2.8686 | 0.3619 | T |
|  | Q model | mrMLM | Chr5_1585607 | 5 | 1585607 | -1.4618 | 3.5647 | 3.7176 | 0.3943 | G |
|  |  | FASTmrMLM | Chr5_1585607 | 5 | 1585607 | -1.1826 | 3.6828 | 2.4396 | 0.3948 | G |
|  |  | FASTmrEMMA | Chr5_1585607 | 5 | 1585607 | -2.1052 | 3.0444 | 1.8336 | 0.3948 | G |
|  |  | pLARmEB | Chr5_1585607 | 5 | 1585607 | -1.125 | 3.2191 | 2.2075 | 0.3948 | G |
|  |  | pLARmEB | Chr8_508116 | 8 | 508116 | -0.7006 | 3.0538 | 1.1119 | 0.3243 | A |
|  |  | FASTmrEMMA | Chr18_1822019 | 18 | 1822019 | -2.2735 | 3.2781 | 2.1399 | 0.4146 | T |
|  | Q+K model | FASTmrEMMA | Chr5_1585607 | 5 | 1585607 | -2.1052 | 3.0444 | 1.8336 | 0.3948 | G |
|  |  | pLARmEB | Chr5_1585607 | 5 | 1585607 | -1.125 | 3.2191 | 2.2075 | 0.3948 | G |
|  |  | pLARmEB | Chr8_508116 | 8 | 508116 | -0.7006 | 3.0538 | 1.1119 | 0.3243 | A |
|  |  | FASTmrMLM | Chr18_17075749 | 18 | 17075749 | 0.8856 | 3.1606 | 1.2751 | 0.2822 | A |
|  |  | FASTmrEMMA | Chr18_1822019 | 18 | 1822019 | -2.2735 | 3.2781 | 2.1399 | 0.4146 | T |
