# Supplementary material for: Genome-wide association study revealed genomic regions associated with tuber quality traits in water yam (Dioscorea alata L.)
Source: PLoS One. 2026 Feb 4;21(2):e0339978. doi: 10.1371/journal.pone.0339978 (PMC12871974; doi:10.1371/journal.pone.0339978)
Supplement: S2 Table — (DOCX) [file pone.0339978.s002.docx]

**S2 Table.** SNP markers associated with tuber dry matter content in water yam

| Trait name | Model | Method | Marker | Chrom | Pos | QTN effect | LOD score | r2 (%) | MAF | Allele |
| --- | --- | --- | --- | --- | --- | --- | --- | --- | --- | --- |
| DMC | Naive | pLARmEB | Chr1_26562068 | 1 | 26562068 | -0.795 | 3.4834 | 0.5192 | 0.245 | G |
|  |  | pKWmEB | Chr1_26788459 | 1 | 26788459 | -0.9591 | 3.9051 | 0.131 | 0.2338 | A |
|  |  | ISIS EM-BLASSO | Chr1_26288741 | 1 | 26288741 | -1.0302 | 4.2064 | 0 | 0.2339 | A |
|  |  | mrMLM | Chr5_22193453 | 5 | 22193453 | 1.1887 | 4.1704 | 1.938 | 0.2649 | C |
|  |  | FASTmrMLM | Chr5_22193453 | 5 | 22193453 | 0.9881 | 4.3947 | 1.3392 | 0.2649 | C |
|  |  | FASTmrEMMA | Chr5_22344109 | 5 | 22344109 | 1.9827 | 3.6876 | 2.5719 | 0.229 | A |
|  |  | pLARmEB | Chr5_22193453 | 5 | 22193453 | 1.117 | 5.4259 | 1.7114 | 0.2649 | C |
|  |  | pKWmEB | Chr5_22193453 | 5 | 22193453 | 1.0685 | 4.8542 | 2.1931 | 0.2649 | C |
|  |  | ISIS EM-BLASSO | Chr5_22344109 | 5 | 22344109 | 1.0616 | 4.5038 | 1.1051 | 0.229 | A |
|  |  | mrMLM | Chr14_19089185 | 14 | 19089185 | 1.4011 | 12.7902 | 14.3351 | 0.4104 | A |
|  |  | FASTmrMLM | Chr14_19089185 | 14 | 19089185 | 1.3487 | 12.0165 | 13.2829 | 0.4084 | A |
|  |  | FASTmrEMMA | Chr14_19089185 | 14 | 19089185 | 2.7379 | 6.1037 | 12.3297 | 0.4084 | A |
|  |  | pLARmEB | Chr14_19089185 | 14 | 19089185 | 1.3015 | 12.1321 | 12.3693 | 0.4084 | A |
|  |  | pKWmEB | Chr14_19089185 | 14 | 19089185 | 1.3566 | 12.4462 | 14.5265 | 0.4104 | A |
|  |  | ISIS EM-BLASSO | Chr14_19089185 | 14 | 19089185 | 1.2652 | 10.8674 | 11.6897 | 0.4084 | A |
|  | Q model | pLARmEB | Chr1_26562068 | 1 | 26562068 | -0.8044 | 3.4488 | 0.5315 | 0.245 | G |
|  |  | ISIS EM-BLASSO | Chr1_26562068 | 1 | 26562068 | -0.8841 | 3.8414 | 0.6421 | 0.245 | G |
|  |  | pLARmEB | Chr5_22193453 | 5 | 22193453 | 0.9326 | 4.0894 | 1.1928 | 0.2649 | C |
|  |  | ISIS EM-BLASSO | Chr5_22344109 | 5 | 22344109 | 0.7977 | 3.0794 | 0.6241 | 0.229 | A |
|  |  | mrMLM | Chr14_19089185 | 14 | 19089185 | 1.3887 | 9.1903 | 14.0821 | 0.4104 | A |
|  |  | FASTmrMLM | Chr14_19089185 | 14 | 19089185 | 1.3094 | 8.3805 | 12.52 | 0.4084 | A |
|  |  | FASTmrEMMA | Chr14_19089185 | 14 | 19089185 | 1.9784 | 5.1221 | 6.4374 | 0.4084 | A |
|  |  | pLARmEB | Chr14_19089185 | 14 | 19089185 | 1.1719 | 6.8937 | 10.0295 | 0.4084 | A |
|  |  | pKWmEB | Chr14_19089185 | 14 | 19089185 | 1.0228 | 5.5257 | 14.3857 | 0.4104 | A |
|  |  | ISIS EM-BLASSO | Chr14_19089185 | 14 | 19089185 | 1.1859 | 6.7594 | 10.2696 | 0.4084 | A |
|  |  | mrMLM | Chr20_16607018 | 20 | 16607018 | -1.3452 | 3.6418 | 3.8403 | 0.2749 | C |
|  |  | FASTmrMLM | Chr20_16607018 | 20 | 16607018 | -0.9869 | 3.6758 | 2.0669 | 0.2735 | C |
|  |  | pKWmEB | Chr20_16607018 | 20 | 16607018 | -1.2842 | 6.0653 | 3.9231 | 0.2749 | C |
|  |  | ISIS EM-BLASSO | Chr20_16607018 | 20 | 16607018 | -0.9403 | 4.0054 | 1.8763 | 0.2735 | C |
|  | Q+K model | FASTmrEMMA | Chr1_26788459 | 1 | 26788459 | -1.8485 | 3.2917 | 1.8462 | 0.2351 | A |
|  |  | pLARmEB | Chr1_26562068 | 1 | 26562068 | -0.8044 | 3.4488 | 0.5315 | 0.245 | G |
|  |  | pKWmEB | Chr1_26562068 | 1 | 26562068 | -0.8495 | 4.5959 | 0.9183 | 0.2438 | G |
|  |  | ISIS EM-BLASSO | Chr1_26562068 | 1 | 26562068 | -0.8841 | 3.8414 | 0.6421 | 0.245 | G |
|  |  | pLARmEB | Chr5_22193453 | 5 | 22193453 | 0.9326 | 4.0894 | 1.1928 | 0.2649 | C |
|  |  | ISIS EM-BLASSO | Chr5_22344109 | 5 | 22344109 | 0.7977 | 3.0794 | 0.6241 | 0.229 | A |
|  |  | FASTmrMLM | Chr14_19089185 | 14 | 19089185 | 1.1665 | 6.0639 | 9.9362 | 0.4084 | A |
|  |  | FASTmrEMMA | Chr14_19089185 | 14 | 19089185 | 2.0812 | 5.7888 | 7.124 | 0.4084 | A |
|  |  | pLARmEB | Chr14_19089185 | 14 | 19089185 | 1.1719 | 6.8937 | 10.0295 | 0.4084 | A |
|  |  | pKWmEB | Chr14_19089185 | 14 | 19089185 | 0.9676 | 7.9157 | 12.7635 | 0.4104 | A |
|  |  | ISIS EM-BLASSO | Chr14_19089185 | 14 | 19089185 | 1.1859 | 6.7594 | 10.2696 | 0.4084 | A |
|  |  | FASTmrMLM | Chr20_16607018 | 20 | 16607018 | -1.1734 | 5.3958 | 2.9221 | 0.2735 | C |
|  |  | pKWmEB | Chr20_16607018 | 20 | 16607018 | -1.1824 | 5.5614 | 3.981 | 0.2749 | C |
|  |  | ISIS EM-BLASSO | Chr20_16607018 | 20 | 16607018 | -0.9403 | 4.0054 | 1.8763 | 0.2735 | C |
