# Supplementary material for: Genome-wide association study revealed genomic regions associated with tuber quality traits in water yam (Dioscorea alata L.)
Source: PLoS One. 2026 Feb 4;21(2):e0339978. doi: 10.1371/journal.pone.0339978 (PMC12871974; doi:10.1371/journal.pone.0339978)
Supplement: S3 Table — (DOCX) [file pone.0339978.s003.docx]

**S3 Table.** SNP markers associated with tuber boiled quality in water yam

| Trait name | Model | Method | Marker | Chrom | Pos | QTN effect | LOD score | r2 (%) | MAF | Allele |
| --- | --- | --- | --- | --- | --- | --- | --- | --- | --- | --- |
| BldT | Naive | pKWmEB | Chr3_753640 | 3 | 753640 | -0.2898 | 3.4757 | 0.5089 | 0.1231 | A |
|  |  | pLARmEB | Chr15_22094249 | 15 | 22094249 | 0.2317 | 4.0264 | 2.0613 | 0.3812 | G |
|  |  | FASTmrEMMA | Chr17_522272 | 17 | 522272 | 0.4657 | 3.9494 | 2.1761 | 0.3775 | G |
|  |  | pKWmEB | Chr17_522272 | 17 | 522272 | 0.2216 | 3.6693 | 2.9141 | 0.3769 | G |
|  |  | FASTmrEMMA | Chr19_23975255 | 19 | 23975255 | -0.6508 | 3.3201 | 2.6562 | 0.1287 | C |
|  |  | ISIS EM-BLASSO | Chr19_23975255 | 19 | 23975255 | -2.00E-04 | 3.4846 | 3.43E-08 | 0.1287 | C |
|  |  | FASTmrEMMA | Chr19_3840797 | 19 | 3840797 | 0.2824 | 3.1012 | 1.7136 | 0.3416 | T |
|  |  | pLARmEB | Chr19_3840797 | 19 | 3840797 | 0.1705 | 3.7017 | 2.6153 | 0.3416 | T |
|  |  | pKWmEB | Chr19_3840797 | 19 | 3840797 | 0.1701 | 4.0776 | 3.3728 | 0.342 | T |
|  |  | ISIS EM-BLASSO | Chr19_3840797 | 19 | 3840797 | 0.1701 | 4.2793 | 2.6025 | 0.3416 | T |
|  | Q model | mrMLM | Chr17_434713 | 17 | 434713 | 0.2447 | 3.2788 | 3.3801 | 0.3458 | A |
|  |  | FASTmrMLM | Chr17_434713 | 17 | 434713 | 0.1834 | 3.0207 | 1.8991 | 0.3465 | A |
|  |  | FASTmrEMMA | Chr17_434713 | 17 | 434713 | 0.3639 | 3.2155 | 1.9701 | 0.3465 | A |
|  |  | pLARmEB | Chr17_434713 | 17 | 434713 | 0.1881 | 3.4033 | 1.9791 | 0.3465 | A |
|  |  | ISIS EM-BLASSO | Chr17_434713 | 17 | 434713 | 0.1835 | 3.323 | 1.9013 | 0.3465 | A |
|  |  | mrMLM | Chr19_3840797 | 19 | 3840797 | 0.2201 | 3.8597 | 4.3618 | 0.342 | T |
|  |  | FASTmrMLM | Chr19_3840797 | 19 | 3840797 | 0.1755 | 3.5158 | 2.7722 | 0.3416 | T |
|  |  | FASTmrEMMA | Chr19_3840797 | 19 | 3840797 | 0.3499 | 3.4585 | 2.631 | 0.3416 | T |
|  |  | pLARmEB | Chr19_3840797 | 19 | 3840797 | 0.1925 | 4.6199 | 3.3074 | 0.3416 | T |
|  |  | pKWmEB | Chr19_3840797 | 19 | 3840797 | 0.1616 | 3.74 | 4.3161 | 0.342 | T |
|  |  | ISIS EM-BLASSO | Chr19_3840797 | 19 | 3840797 | 0.1756 | 4.4586 | 2.7753 | 0.3416 | T |
|  | Q+K model | FASTmrMLM | Chr17_434713 | 17 | 434713 | 0.1829 | 3.2788 | 1.8884 | 0.3465 | A |
|  |  | FASTmrEMMA | Chr17_434713 | 17 | 434713 | 0.3639 | 3.2155 | 1.9701 | 0.3465 | A |
|  |  | pLARmEB | Chr17_434713 | 17 | 434713 | 0.1881 | 3.4033 | 1.9791 | 0.3465 | A |
|  |  | ISIS EM-BLASSO | Chr17_434713 | 17 | 434713 | 0.1835 | 3.323 | 1.9013 | 0.3465 | A |
|  |  | pKWmEB | Chr17_522272 | 17 | 522272 | 0.1905 | 3.3756 | 2.5401 | 0.3769 | G |
|  |  | mrMLM | Chr19_3840797 | 19 | 3840797 | 0.2165 | 3.6983 | 4.2169 | 0.342 | T |
|  |  | FASTmrMLM | Chr19_3840797 | 19 | 3840797 | 0.1755 | 3.8597 | 2.7721 | 0.3416 | T |
|  |  | FASTmrEMMA | Chr19_3840797 | 19 | 3840797 | 0.3499 | 3.4585 | 2.631 | 0.3416 | T |
|  |  | pLARmEB | Chr19_3840797 | 19 | 3840797 | 0.1925 | 4.6199 | 3.3074 | 0.3416 | T |
|  |  | pKWmEB | Chr19_3840797 | 19 | 3840797 | 0.1616 | 4.432 | 4.2992 | 0.342 | T |
|  |  | ISIS EM-BLASSO | Chr19_3840797 | 19 | 3840797 | 0.1756 | 4.4586 | 2.7753 | 0.3416 | T |
