# Supplementary material for: Genome-wide association study revealed genomic regions associated with tuber quality traits in water yam (Dioscorea alata L.)
Source: PLoS One. 2026 Feb 4;21(2):e0339978. doi: 10.1371/journal.pone.0339978 (PMC12871974; doi:10.1371/journal.pone.0339978)
Supplement: S4 Table — (DOCX) [file pone.0339978.s004.docx]

**S4 Table.** SNP markers associated with tuber pounded quality in water yam.

| Trait name | Model | Method | Marker | Chrom | Pos | QTN effect | LOD score | r2 (%) | MAF | Allele |
| --- | --- | --- | --- | --- | --- | --- | --- | --- | --- | --- |
| PndT | Naive | pLARmEB | Chr2_21553389 | 2 | 21553389 | -0.166 | 4.8289 | 1.5018 | 0.1881 | C |
|  |  | mrMLM | Chr8_9354121 | 8 | 9354121 | -0.2505 | 4.0893 | 4.1954 | 0.3159 | C |
|  |  | FASTmrMLM | Chr8_9354121 | 8 | 9354121 | -0.2015 | 3.4804 | 2.7452 | 0.3144 | C |
|  |  | mrMLM | Chr14_21319040 | 14 | 21319040 | 0.205 | 3.0134 | 4.2595 | 0.4714 | A |
|  |  | FASTmrMLM | Chr14_21319040 | 14 | 21319040 | 0.1476 | 3.2444 | 2.2356 | 0.4728 | A |
|  |  | FASTmrEMMA | Chr14_21319040 | 14 | 21319040 | 0.2879 | 3.079 | 1.594 | 0.4728 | A |
|  |  | pLARmEB | Chr14_21319040 | 14 | 21319040 | 0.1502 | 3.568 | 2.3124 | 0.4728 | A |
|  |  | pKWmEB | Chr14_21319040 | 14 | 21319040 | 0.1435 | 3.6187 | 3.9313 | 0.4714 | A |
|  |  | ISIS EM-BLASSO | Chr14_21319040 | 14 | 21319040 | 0.1346 | 3.1137 | 1.8582 | 0.4728 | A |
|  | Q model | FASTmrMLM | Chr14_21319040 | 14 | 21319040 | 0.1835 | 4.2105 | 3.4536 | 0.4728 | A |
|  |  | pLARmEB | Chr14_21319040 | 14 | 21319040 | 0.1836 | 4.2105 | 3.4399 | 0.4728 | A |
|  |  | pKWmEB | Chr14_21319040 | 14 | 21319040 | 0.1742 | 3.6762 | 5.5523 | 0.4714 | A |
|  |  | ISIS EM-BLASSO | Chr14_21319040 | 14 | 21319040 | 0.1764 | 3.7265 | 3.1924 | 0.4728 | A |
|  | Q+K model | mrMLM | Chr14_21319040 | 14 | 21319040 | 0.2314 | 3.653 | 5.4907 | 0.4714 | A |
|  |  | FASTmrMLM | Chr14_21319040 | 14 | 21319040 | 0.1835 | 4.2105 | 3.4552 | 0.4728 | A |
|  |  | pLARmEB | Chr14_21319040 | 14 | 21319040 | 0.1836 | 4.2105 | 3.4399 | 0.4728 | A |
|  |  | pKWmEB | Chr14_21319040 | 14 | 21319040 | 0.1743 | 4.0606 | 5.5523 | 0.4714 | A |
|  |  | ISIS EM-BLASSO | Chr14_21319040 | 14 | 21319040 | 0.1764 | 3.7265 | 3.1924 | 0.4728 | A |
