# Supplementary material for: Genome-wide association study revealed genomic regions associated with tuber quality traits in water yam (Dioscorea alata L.)
Source: PLoS One. 2026 Feb 4;21(2):e0339978. doi: 10.1371/journal.pone.0339978 (PMC12871974; doi:10.1371/journal.pone.0339978)
Supplement: S8 Fig — The R2 color key indicates the degree of significant association with the putative genes. (DOCX) [file pone.0339978.s008.docx]

**
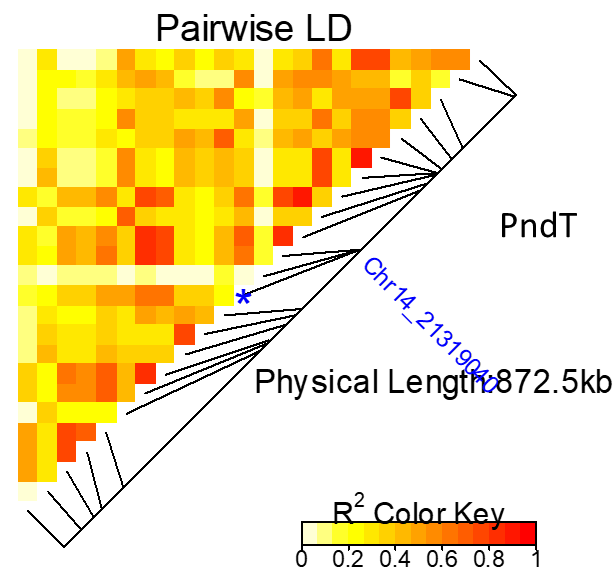
**

S8 Figure. Heatmap LD haplotype blocks for different SNP markers located on different chromosomes associated with PndT.

The R2 color key indicates the degree of significant association with the putative genes
